# Effects of Muscle Energy Technique and Joint Manipulation on Pulmonary Functions, Mobility, Disease Exacerbations, and Health-Related Quality of Life in Chronic Obstructive Pulmonary Disease Patients: A Quasiexperimental Study

**DOI:** 10.1155/2022/5528724

**Published:** 2022-07-30

**Authors:** Diksha Bains, Aksh Chahal, Mohammad Abu Shaphe, Faizan Z. Kashoo, Taimul Ali, Ahmad H. Alghadir, Masood Khan

**Affiliations:** ^1^Maharishi Markandeshwar Institute of Physiotherapy and Rehabilitation, Maharishi Markandeshwar (Deemed to be University), Mullana, Haryana, India; ^2^Department of Physical Therapy, College of Applied Medical Sciences, Jazan University, Jazan, Saudi Arabia; ^3^Department of Physical Therapy and Health Rehabilitation, College of Applied Medical Sciences, Majmaah University, Majmaah, Saudi Arabia; ^4^College of Physiotherapy, Peerless Hospitex Hospital & Research Center, Kolkata, India; ^5^Rehabilitation Research Chair, Department of Rehabilitation Sciences, College of Applied Medical Sciences, King Saud University, Riyadh, Saudi Arabia

## Abstract

Chronic obstructive pulmonary disease (COPD) is primarily a disease of the lungs; however, extrapulmonary comorbidities like rib cage stiffness, decreased thoracic spine mobility, postural changes, and skeletal muscle dysfunctions also coexist. Muscle energy technique (MET) and joint manipulation (JM) may help alleviate these musculoskeletal problems. This study was aimed at evaluating the effectiveness of MET and JM on pulmonary functions, dyspnea, chest wall mobility, disease exacerbations, and health-related quality of life in COPD patients. A total of 16 patients (7 women and 9 men) suffering from COPD between the ages of 35 and 65 years participated in the study. Pretest-posttest quasiexperimental design was used. MET was applied to the sternocleidomastoid, anterior scalene, pectoralis major muscles, and at the C4-C6 level of the cervical spine. Maitland JM was performed in the thoracic region at the intervertebral, costovertebral, and costotransverse joints. The treatment intervention lasted for 3 weeks. FEV_1_/FVC, maximum inspiratory pressure (MIP), SpO2, modified Borg dyspnea scale (MBDS), COPD assessment test (CAT), mMRC dyspnea scale, BODE index, right and left hemidiaphragm excursion, and chest wall expansion at T4 and T10 levels were the outcome measures. Significant improvement (*p* < 0.05) was observed in FEV_1_/FVC, MIP, SpO2, MBDS, CAT, mMRC dyspnea scale, BODE index, and chest expansion at T4 and T10 levels. Only for the hemidiaphragm excursion, no significant (*p* > 0.05) improvement was observed. Combined application of MET to accessory respiratory muscles and cervical spine and JM to thoracic spine improved pulmonary functions, chest wall mobility, and health-related quality of life and reduced dyspnea and disease exacerbations in patients with mild to moderate COPD.

## 1. Introduction

Chronic obstructive pulmonary disease (COPD) is among the leading causes of mortality and morbidity in low-, middle, and high-income countries [1]. In COPD, obstruction or limitation in airflow occurs due to emphysema (parenchymal destruction), a mixture of small airway diseases, and in many cases asthma (increased airway responsiveness) [1]. In addition to the involvement of the lungs in COPD, there are also extrapulmonary comorbidities [2, 3]. These extrapulmonary comorbidities may include stiffness of the rib cage [4], reduced spinal motion [5], increased muscle sensitivity [5], postural changes [5], cervicothoracic pain, muscle loss, osteoporosis, and/or skeletal muscle dysfunction [6, 7]. The activity-limiting dyspnea that occurs in COPD patients may be caused by mechanical restrictions [8, 9]. Reduced thoracic axial rotation and altered neck posture in COPD patients were found to be associated with poorer pulmonary functions [5]. There is evidence to show that pathophysiological changes in COPD are related to the inflammatory status and oxidative stress that occurs in COPD [10]. Skeletal muscle wasting and weight loss in COPD patients have been proposed to be related to the imbalance of oxidative stress status [11, 12]. One recent study concluded that COPD-related sarcopenia is related to increased oxidative stress-related factors [13].

Regarding the treatment of COPD, international guidelines recommend that the management and treatment of COPD should be individualized to reduce symptoms, improve exercise tolerance and quality of life, and reduce the chances of exacerbations [3, 14]. Physical therapy has an important role to play in addressing musculoskeletal disorders in COPD patients.

Previous studies have evaluated the use of manipulative osteopathic treatments [15, 16], soft tissue techniques [17], myofascial release techniques [18], and spinal joint manipulation (JM) [19, 20] in the treatment of patients with COPD with varying results. Muscle energy technique (MET) and spinal JM are used to treat musculoskeletal problems in patients with COPD. MET is a form of manual therapy in which the patient performs voluntary contraction against the counterforce applied directly by the therapist [21]. MET is used to increase the length of the spastic, shortened, or contractured muscles. Localized edema can also be reduced with MET by muscle pump action. MET can also increase the strength of physiologically weakened muscles [21] and can be used to mobilize articulation whose mobility is reduced [22]. Previous studies have shown that MET can increase shoulder range of motion (ROM) [23, 24], spinal ROM [25, 26], and muscle flexibility [27, 28]. Another technique, spinal manipulative therapy (high-grade JM), was found to improve chest wall compliance when applied to paravertebral tissues or the region of spinal stiffness [29]. JM of the spine consists of high-velocity low-amplitude thrust to the thoracic intervertebral, costovertebral, and costotransverse joints. Spinal JM is hypothesized to decrease the rigidity of the chest wall and increase the mobility of the costal and spinal joints [30]. However, a systemic review reported that there was insufficient evidence to support or refute the use of manual therapy in the treatment of COPD [31].

Due to the pathophysiology of COPD, where several musculoskeletal dysfunctions coexist, MET or spinal JM alone may not be able to provide desirable improvements. If both techniques are applied to patients, which is conveniently possible in clinical settings, then we may get better results.

To our knowledge, no study has examined the effects of MET and JM, when applied together, on lung functions, dyspnea, chest wall mobility, disease exacerbations, and health-related quality of life in patients with COPD. Therefore, a study was warranted that examined the cumulative effects of MET and JM. The present study was aimed at assessing the effects of MET and JM on pulmonary functions, dyspnea, chest wall mobility, disease exacerbations, and health-related quality of life in patients with COPD. We hypothesized that MET and JM when applied together improve pulmonary functions, chest wall mobility, and health-related quality of life and reduce dyspnea and disease exacerbations, in patients with COPD.

## 2. Materials and Methods

### 2.1. Study Design and Participants

A single-group pretest-posttest quasiexperimental design was used. Due to the COVID-19 pandemic, COPD patients were not easily available; therefore, a convenient sampling method was performed. In retrospect, the minimum sample size was calculated to be 12 for a quasiexperimental study, using the software G∗Power 3.1.9.4. (Heinrich-Heine-Universität Düsseldorf, Düsseldorf, Germany; http://www.gpower.hhu.de/), from the data obtained in the present study (effect *size* = 0.99; *α* = 0.05; power (1 − *β*) = 0.80; Wilcoxon signed-rank test). A total of 22 participants aged 35-65 years were screened for the study; however, two participants declined to participate and four participants could not complete the 3-week protocol; therefore, data of the 16 participants were analyzed. Patients diagnosed with COPD were recruited from the Department of Respiratory Medicine of the tertiary care hospital and referred to the Musculoskeletal Physiotherapy Research Laboratory. The selected participants were currently nonsmokers and had *FEV*1/*FVC* < 0.70 and oxygen saturation of >95%. Patients diagnosed with severe and very severe COPD, osteoporosis, thoracic joint instability, scoliosis, neurological disease, cardiovascular disease, cognitive disorder, recent abdominal or chest surgery, pneumothorax, haemothorax, tuberculosis, pneumonia, lung carcinoma, and high anxiety level related to treatment were excluded from the study. This study was prospectively registered before recruiting participants in the Indian Clinical Trial Registry with the registration number CTRI/2020/04/024648 and obtained its Universal Trial Number U1111-1247-6630. The protocol copyright related to the study was registered with the unique registration number L-97600/2020 under the Copyright Office of the Government of India. The risks and benefits of the study were discussed with all participants who participated voluntarily, and informed consent was obtained. The study was conducted in accordance with the Declaration of Helsinki and approved by the Institutional Research Ethics Committee at Maharishi Markandeshwar (deemed to be University), Mullana, Ambala (Protocol ID: MMDU/IEC-1547 and 10 December, 2019).

### 2.2. Outcome Measures

#### 2.2.1. Primary

- FEV_1_/FVC ratio measured by spirometer [32]

#### 2.2.2. Secondary

- Maximum inspiratory pressure (MIP), measured by a portable capsule sensing pressure gauge [33]
- SpO2, measured by pulse oximeter [34]
- Modified Borg dyspnea scale (MBDS) [35]
- COPD assessment test (CAT) [36]
- mMRC dyspnea scale (modified Medical Research Council) [37, 38]
- BODE index (body mass index, airflow obstruction, dyspnea, and exercise) [39]
- Right and left hemidiaphragm excursion measured by chest radiograph [40]
- The expansion of the chest wall was measured at the level of the fourth thoracic vertebra (T4) and the tenth thoracic vertebra (T10) using a measuring tape

### 2.3. Instrumentation

- RMS PC-Based Spirometer Helios-401 (Recorders & Medicare Systems Pvt. Ltd., Haryana, India) [41]
- Portable capsule sensing pressure gauge (Gauges Bourdon (India) Pvt. Ltd., New Delhi, India) [33]
- Pulse oximeter (Choicemmed MD300C2, China) [42]
- Chest radiographs [43]
- Measuring tape [44]

### 2.4. Study Protocol

The study protocol was divided into three phases.

#### 2.4.1. Preintervention Evaluation

The baseline measurement of all primary and secondary outcome measures was taken before the application of the intervention.

Lung volumes (FEV_1_ and FVC): in the sitting position, participants were asked to first inhale as deeply as possible and then exhale from the mouth into the spirometer tube as forcefully as they could. Then, the exhaled volume of air in the first second (FEV_1_) [45] and total volume of air exhaled (FVC) [46] were recorded. FEV_1_ was also used for the calculation of the BODE index

MIP: participants were made to sit comfortably, and then, a nose clip was applied to the participants' nose to avoid air leaks. They were asked to hold the gauge with both hands and close their lips firmly around the mouthpiece. Then, they were asked to exhale as much as possible and then to inhale maximally for more than 1 sec against the resistance of the gauge. MIP reading in the portable capsule sensing pressure gauge was recorded [47]

MBDS/CAT/mMRC scale: each participant was asked to complete these scales

SpO2: a fingertip pulse oximeter was used to measure SpO2 [48]

6-minute walk distance for BODE index: each participant was asked to walk as far as possible for 6 minutes [49]. The distance walked was measured in meters and used to calculate the BODE index

Hemidiaphragm excursion: excursion of the right and left hemidiaphragm was measured by anteroposterior chest radiographs in the supine position. A radiopaque ruler was placed on the chest and abdominal area of the participants in the midline in the craniocaudal direction. X-ray films were obtained during maximum inspiration and expiration. Then, the distance between the two levels of both hemidiaphragm was noted [50]

Chest wall expansion: the participants stood with feet 5 cm apart and arms elevated. Chest wall expansion was measured at two levels, upper and lower. For the upper level, a measuring tape was placed around the chest at the T4 spinous process and the fourth intercostal space. For the lower level, a measuring tape was placed at the T10 spinous process and the xiphoid process. The participants were asked to maximally inhale and exhale. The difference between these two extremes was noted [51]

#### 2.4.2. Intervention

MET followed by JM was applied to all participants. MET was applied to the following muscles and regions: sternocleidomastoid (SCM), anterior scalene, pectoralis major, and at the C4-C6 level of the cervical spine. Grade V (high velocity, low amplitude) Maitland JM was performed in the thoracic region at the intervertebral, costovertebral, and costotransverse joints. This intervention was carried out twice a week for a total of 3 weeks.

*(1) MET*. For SCM, the participants were made to lie in a supine position with arms on their sides. The physical therapist (PT) performed stretching of SCM with his arms crossed and hands stabilized the participant's mastoid area and shoulder. The participants were asked to perform the action of SCM with 20% of the maximum strength, from both ends against the resistance of PT. The participants put effort for 7-10 seconds followed by relaxation, and then, the PT took it to the new barrier to increase the degree of side bending and rotation, where it was stabilized, and then, the shoulder was stretched caudally. Once the muscle was in a stretched position, the patient relaxed, and the stretch was held for up to 30 seconds [22].

For the anterior scalene muscle, the participants were made to lie supine with a cushion or towel under the upper thoracic area. The PT placed his hand on the side of the participant's face/forehead to resist the isometric contraction and the other hand on the sternum. The participants were asked to perform the action of the anterior scalene muscle against PT resistance and hold it for 7-8 seconds followed by relaxation [22].

For pectoralis major, the participants were supine and the PT was on the ipsilateral side. The PT placed one hand on the sternum and applied the lateral compression force, placed another hand on the anterior shoulder, and applied the force in the posterolateral direction. Then, the participants were asked to exert force in the anterior direction towards the ceiling for 5-7 seconds, followed by relaxation, and then take it to the new barrier by taking up the slack 2-3 times [22].

For the cervical spine (C4-C6), in the supine position, the neck of the participants was slightly flexed, completely bent on the side, and rotated to the ipsilateral side. The middle fingers of the PT's right hand were placed over the pillars of C4-C6. The PT placed his other hand on the left parietal and temporal area of the patient. The participants were asked to bend and rotate the neck towards the contralateral side against the resistance of the PT, for 5-7 seconds, followed by relaxation, and then taken to its new barrier, and the same procedure was repeated 2-3 times [22].

*(2) Joint Manipulation (Thoracic Spine)*. The participants were made to lie in the prone position. The PT placed his hands parallel to each other on both sides of the participants' thoracic spine over their back. One hand was placed caudal and another cephalad to the joints to be mobilized. Then, the PT applied the posteroanterior and rotational component with the right hand towards the caudal direction and with the left hand towards the lateral and cephalad direction. The technique was performed rhythmically along with the participant's breathing pattern, and the manipulative thrust was administered at the end of the expiration. This technique consisted of oscillatory movements applied in three directions: posteroanterior, caudal, and lateral. This manipulation mobilized the intervertebral, costovertebral, and costotransverse joints [52].

#### 2.4.3. Postintervention Evaluation

All outcome measures were measured again after a 3-week intervention similar to the case of preintervention evaluation.

### 2.5. Data Analysis

Data analysis was performed using SPSS version 26 statistical software (SPSS Inc., Chicago, IL, USA). The Shapiro-Wilk normality test was used to assess the normal distribution of the data. For normally distributed data, a paired *t*-test was used. The paired *t*-test is a parametric test, used to test if the means of two paired measurements (e.g., pretest/posttest) are significantly different [53]. For ordinal and not normally distributed data, the Wilcoxon signed-rank test was used. The Wilcoxon signed-rank test is a nonparametric test used as an alternative to the paired Student's *t*-test. This test does not assume that the samples are normally distributed [54]. The confidence interval was established at 95%, and the *p* value < 0.05 was considered significant. For variables having normal distribution, arithmetic mean was used; however for variables that did not have a normal distribution, geometric mean was used.

## 3. Results and Discussion

Data from 16 participants were analyzed.  Table 1 includes some demographic characteristics of all participants.

Wilcoxon signed-rank test: this test was performed for the following variables: FEV_1_/FVC, MBDS, BODE index, mMRC dyspnea scale, SpO2, CAT (COPD assessment test), and hemidiaphragm excursion (right and left). Significant improvement (*p* < 0.05) was observed in all variables except for both hemidiaphragm excursions (right and left) (Table 2). For significant values, Cohen's *d* showed a large effect size

Paired *t*-test: this test was performed for the following variables: MIP and chest expansion at levels T4 and T10. A significant improvement was observed in all variables (Table 3). For significant values, Cohen's *d* showed a large effect size

The present study was aimed at evaluating the effectiveness of MET and JM when applied together, on pulmonary functions, MIP, SpO2, dyspnea, diaphragm excursion, disease exacerbations, chest wall mobility, and health-related quality of life in patients with COPD. The results of the present study showed that application of MET (on accessory respiratory muscles and C4-C6 spine) and JM (on thoracic spine joints) improved spirometry (FEV_1_/FVC ratio), MIP, SpO2, chest wall mobility, and health-related quality of life and reduced dyspnea and disease exacerbations in patients with COPD.

In the current study, geometric mean (GM) was calculated for nonparametric variables as the values were altered by the outliers, and the mean of data tended to make large fluctuations. Thus, GM gave an appropriate mean of the data set by neglecting the factors that provided values in negative and zero and obstructed the mean data. In the case of a skewed distribution of the data, by GM, the symmetry of data was made by log transformation [55]. The GM provides values less than the actual arithmetic mean, as the arithmetic mean gives a sum of the total number of values and is sensitive to outliers, while the effect of outliers on the geometric mean is mild [55]. Thus, in the case of a nonparametric test, the exact mean was obtained by GM.

To the best of our knowledge, no study has used both MET and JM simultaneously in COPD patients. Therefore, it is difficult to compare this study with the previous studies. However, several previous studies have used either MET or JM along with other interventions for the management of COPD patients. One of the previous studies in COPD patients reported an improvement in lung function after the application of MET to the accessory respiratory muscles in conjunction with other soft tissue manual therapy techniques [17]. The study by Putt et al. [23] reported an increase in lung capacity in COPD patients after applying the hold-relax technique (PNF) to the pectoralis major muscle. Since the PNF technique and MET are similar in their principle of stretching and facilitating joints and muscles [56], therefore, the study by Putt et al. [23] supports the findings of the present study.

In the current work, improvement was observed in dyspnea scales (MBDS, mMRC dyspnea scale, and BODE index) also. The mechanism behind this improvement can be explained as follows: there is a feeling of breathlessness in patients with COPD, and to overcome this feeling, the patients continuously use accessory respiratory muscles, leading to shortening and tightening of these muscles [57]. MET stretching of these accessory muscles relaxes them and reduces the rate of muscle spindle firing in the lengthening phase. Due to this, the central respiratory motor command required for the given ventilation is decreased; thus, as a result, dyspnea may be alleviated [58]. MET corrects respiratory mechanics by correcting accessory inspiratory muscle dysfunctions; thus, diaphragmatic breathing is made more effective. This increases ventilation, which increases V/Q matching, resulting in an improvement in SpO2 levels [56]. This may explain the increase in SpO2 level in the present study.

The present study showed an improvement in the BODE index, which includes the BMI, FEV_1_, mMRC scale, and the distance walked in 6 minutes. Marin et al. reported that dyspnea (modified MRC scale) was a good predictor of walking distance in their study [59]. Therefore, alleviating dyspnea and improving SpO2 using the mechanisms mentioned above will explain the improved BODE index through an increase in walking distance in the 6-minute walk test, improved FEV_1_/FVC, and improved mMRC scores. The BODE index is reported to be a good predictor of mortality in COPD patients, in the medium to long term [60]. Therefore, the improvement in the BODE index after the application of MET and JM in the present study is significant from a functional status perspective of this population.

Spinal JM (high velocity, low amplitude) has been reported to increase spinal ROM [61] and decrease local hypertonicity of the muscles [62]. Previous studies in normal individuals have reported that increased mobility of the thoracic joints improved lung functions in the short term [63, 64]. Therefore, in the present study, manipulation of the thoracic joints may have increased mobility of the thoracic spine, which in turn may have resulted in increased lung functions.

In the present study, minimal clinically important difference (MCID) values were also calculated for comparison, but only for the variables whose MCID values were already provided in previous studies/literature. For FEV_1_/FVC and MIP, MCID could not be found in the literature. For MBDS, a previous study reported an MCID value of 1 unit [65]. In the present study, MCID and standard error of measurement (SEM) for MBDS were found to be 2.536 and 0.915, respectively; therefore, both clinically and statistically significant improvements were found. For chest expansion, a previous study reported that MCID change scores should be greater than 3.60 for the upper chest and 4.40 for the lower chest expansion [66]. In the present study, the MCID for the upper (T4) and lower (T10) chest expansions was 0.684 and 0.554, respectively, and the SEM for the upper (T4) and lower (T10) chest expansion was 0.247 and 0.2, respectively. Therefore, no clinically significant differences were found in the chest expansion in the present study after the application of the intervention. The previous study has cited ±4 percentage points as MCID for SpO2 [67]. In the present study, MCID and SEM were 6.5% and 0.237, respectively. Therefore, for SpO2, statistically and clinically significant results were found. MCID for CAT is reported to be a change of 2 points [68]. The present study found MCID and SEM for CAT to be 3.045 and 1.099, respectively. Therefore, in the present study, for CAT, statistically and clinically significant results were obtained. A previous study reported MCID for mMRC as 1 [69]. In the present study, MCID and SEM for mMRC were found to be 0.70 and 0.25, showing that statistically and clinically significant results were observed on the mMRC dyspnea scale after the application of MET and JM.

No improvement was observed in diaphragmatic excursion in the present study. One of the recent studies by Jung et al. [70] reported improvement in diaphragmatic excursion after 8 weeks of thoracic mobilization in individuals with thoracic hyperkyphosis. The reason why there is no significant change in diaphragmatic excursion in the present study may be the short duration of the intervention. A longer duration intervention (8 weeks) may have brought the desired changes in diaphragmatic excursion.

The present study has several limitations also. Due to the limited availability of patients with COPD, no control group could be included in the study. Therefore, the lack of a control group limits the comparison of participants who received the intervention (MET and JM) with those who did not receive the same treatment during the same period. The present study did not include long-term follow-up; therefore, the improvements observed with the intervention may be temporary and short-lived. Therefore, future research is needed that includes a large sample size, a control group, and long-term follow-up. It may be possible that of the two interventions (MET and JM), only one of them is sufficient to bring about the desired improvements. Therefore, further studies should also evaluate the efficacy of MET alone versus JM alone in COPD patients.

## 4. Conclusions

Combined application of MET to accessory respiratory muscles and cervical spine and JM to thoracic spine (intervertebral, costovertebral, and costotransverse joints) improved pulmonary functions, chest wall mobility, and health-related quality of life and reduced dyspnea and disease exacerbations in patients with mild to moderate COPD. Therefore, a combination of MET and JM can be used as a physiotherapeutic intervention to improve the above-mentioned outcome measures in patients with COPD. These techniques can be an adjunct to breathing exercises and positioning techniques (postural drainage) to relieve symptoms and achieve a better quality of life in this population group; however, further experimental trials are needed to verify this claim.

## Figures and Tables

**Table 1:** Demographic characteristics of the participants (*n* = 16), mean (LL-UL).

	Mean (LL-UL)
Age (years)	49.25 (43.70-54.80)
Height (meter)	1.65 (1.60-1.70)
Body mass (kg)	62.2 (56.4-67.98)
BMI (kg/m^2^)	23.07 (20.7-25.5)
Male/female	9/7

LL: lower limit; UL: upper limit; BMI: body mass index.

**Table 2:** Dependent variable data, AM (LL-UL) and GM at baseline and postintervention, Wilcoxon signed-rank test *p* values, and Cohen's *d* values.

Variables	Baseline		Post-intervention		*p* value	Cohen's *d*
	AM (LL-UL)	GM	AM (LL-UL)	GM		
FEV_1_/FVC (%)	57.94 (54.1-61.8)	57.49	68.88 (63.16-74.59)	68.17	0.001^∗^	0.99
MBDS (n)	4.38 (3.95-4.80)	4.30	2.87 (2.45-3.30)	2.75	<0.001^∗^	0.99
BODE (n)	6.0 (5.3-6.7)	5.88	3.8 (3.13-4.5)	3.56	0.002^∗^	0.99
mMRC (n)	2.06 (1.70-2.42)	1.943	0.875 (0.60-1.14)	1.054	<0.001^∗^	0.99
SpO2 (%)	98.50 (98.2-98.8)	98.49	99.3 (99.0-99.6)	99.31	<0.001^∗^	0.99
CAT (*n*)	11.5 (10.5-12.5)	11.35	6.06 (5.4-6.7)	5.94	<0.001^∗^	1.00
Left hemidiaphragm excursion (cm)	1.5 (1.45-1.6)	1.52	1.55 (1.5-1.6)	1.54	0.157	0.6
Right hemidiaphragm excursion (cm)	1.7 (1.6-1.8)	1.67	1.7 (1.6-1.74)	1.66	0.480	0.58

∗Significant (*p* < 0.05). AM: arithmetic mean; LL: lower limit; UL: upper limit; GM: geometric mean; FEV_1_: forced expiratory volume in one second; FVC: forced vital capacity; MBDS: modified Borg dyspnea scale; BODE: body mass index, airflow obstruction, dyspnea, and exercise; mMRC: modified Medical Research Council dyspnea scale; SpO2: peripheral capillary oxygen saturation; CAT: chronic obstructive pulmonary disease evaluation test.

**Table 3:** Dependent variables data, mean (LL-UL) at baseline and postintervention, paired *t*-test *p* values, and Cohen's *d* values.

Variables	Mean (LL-UL)		*t* value	*p* value	Cohen's *d*
	Baseline	Postintervention			
MIP (cmH_2_O)	42.56 (37.01-48.11)	49.37 (44.33-54.41)	-8.662	<0.001^∗^	1.00
Chest expansion (cm) (T4)	2.4 (2.0-2.7)	2.6 (2.3-2.9)	-5.614	<0.001^∗^	1.00
Chest expansion (cm) (T10)	2.5 (2.2-2.8)	2.7 (2.5-2.98)	-4.772	<0.001^∗^	1.00

∗Significant (*p* < 0.05). LL: lower limit; UL: upper limit; MIP: maximum inspiratory pressure; T4: fourth thoracic vertebra; T10: tenth thoracic vertebra.

## Data Availability

The data associated with the paper are not publicly available but are available from the corresponding author on reasonable request.
